# Spatial Confinement of Pt Nanoparticles in Carbon Nanotubes for Efficient and Selective H_2_ Evolution from Methanol

**DOI:** 10.1002/advs.202306893

**Published:** 2024-01-15

**Authors:** Xiaotao Jin, Jiaying Yan, Xiang Liu, Qing Zhang, Yingping Huang, Yanlan Wang, Changlong Wang, Yufeng Wu

**Affiliations:** ^1^ Engineering Research Center of Eco‐environment in Three Gorges Reservoir Region College of Materials and Chemical Engineering China Three Gorges University Yichang Hubei 443002 P. R. China; ^2^ Department of chemistry and chemical engineering Liaocheng University Liaocheng 252059 P. R. China; ^3^ Institute of Circular Economy, Faculty of Materials and Manufacturing Beijing University of Technology Beijing 100124 P. R. China

**Keywords:** hydrogen, KIE, methanol steam reforming, Pt/CNT, tandem reaction

## Abstract

H_2_ generation from methanol‐water mixtures often requires high pressure and high temperature (200–300 °C). However, CO can be easily generated and poison the catalytic system under such high temperature. Therefore, it is highly desirable to develop the efficient catalytic systems for H_2_ production from methanol at room temperature, even at sub‐zero temperatures. Herein, carbon nanotube‐supported Pt nanocomposites are designed and synthesized as high‐performance nano‐catalysts, via stabilization of Pt nanoparticles onto carbon nanotube (CNT), for H_2_ production upon methanol dehydrogenation at sub‐zero temperatures. Therein, the optimal Pt/CNT nanocomposite presents the superior catalytic performance in H_2_ production upon methanol dehydrogenation at the expense of B_2_(OH)_4_, with the TOF of 299.51 min^‐1^30 ^o^C. Compared with other common carriers, Pt/CNT exhibited the highest catalytic performance in H_2_ production, emphasizing the critical role of CNT in methanol dehydrogenation. The confinement of Pt nanoparticles by CNTs is conducive to inhibiting the aggregation of Pt nanoparticles, thereby significantly increasing its catalytic performance and stability. The kinetic study, detailed mechanistic insights, and density functional theory (DFT) calculation confirm that the breaking of O─H bond of CH_3_OH is the rate‐controlling step for methanol dehydrogenation, and both H atoms of H_2_ are supplied by methanol. Interestingly, H_2_ is also successfully produced from methanol dehydrogenation at −10 °C, which absolutely solves the freezing problem in the H_2_ evolution upon water‐splitting reaction.

## Graphical abstract

Carbon nanotube‐supported Pt, Pd, and Rh nanocomposites (Pt/CNT, Pd/CNT, and Rh/CNT) are designed and synthesized as high‐performance nano‐catalysts, via spatial confinement of Pt, Pd, and Rh nanoparticles into CNT, for H2 production upon methanol dehydrogenation in sub‐zero temperatures.

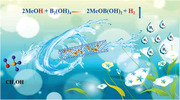

## 1 Introduction

Nowadays, the world's development of economy and population experiences a rapid increase, provoking an explosive growth in energy consumption.^[^ ^1^, ^2^, ^3^, ^4^, ^5^ ^]^ The excessive consumption of traditional fossil energy leads to global warming and environmental problems.^[^ ^6^, ^7^, ^8^, ^9^, ^10^ ^]^ Hence, it is highly desired to develop sustainable, green and carbon‐free energy. H_2_ is deemed as the most prospective alternative for traditional fossil energy because of its high calorific value, zero‐emission, and easy accessibility.^[^ ^11^, ^12^, ^13^, ^14^, ^15^ ^]^ In general, the industrial H_2_ generation (95%) is mainly achieved by coal gasification (C + 2H_2_O → CO_2_ + 2H_2_) and methane steam reforming (CH_4_ + 2H_2_O → CO_2_ + 4H_2_).^[^ ^16^, ^17^, ^18^, ^19^, ^20^, ^21^ ^]^ It is obvious that major H_2_ generation methods rely heavily on conventional fossil fuels. This is a complete departure from the principles of the utilization of hydrogen energy.^[^ ^22^ ^]^ Additionally, the large‐scale industrial application of hydrogen is severely hampered by its safety issues as to storing and transportation, because of its extremely low density, super‐high explosibility, and liquefaction dilemma.^[^ ^23^ ^]^ Therefore, it still remains a severe challenge to develop hydrogen storage materials, including HCOOH,^[^ ^24^, ^25^, ^26^, ^27^ ^]^ hydrazine,^[^ ^28^ ^]^ borohydrides^[^ ^29^, ^30^ ^]^ and alcohols,^[^ ^31^ ^]^ for avoiding high cost and the safety risks in the production, storage, and transportation of hydrogen.

Methanol, which can be produced from biomass on a large scale, has been regarded as a potential hydrogen source due to its low‐cost and super‐high hydrogen density (12.5 wt.%).^[^ ^31^, ^32^, ^33^, ^34^ ^]^ In fact, H_2_ generation from methanol‐water mixtures (CH_3_OH + H_2_O → 3H_2_ + CO_2_), which is also called as methanol steam reforming, has been well developed and widely used in the vehicular power generation.^[^ ^35^, ^36^, ^37^ ^]^ Homogeneous catalysts for H_2_ production from methanol are developed for ages, but they exhibited poor activity and selectivity.^[^ ^38^ ^]^ Indeed, some low molecular weight organic matter (such as acetic acid,^[^ ^39^ ^]^ formaldehyed,^[^ ^40^ ^]^ formate salts,^[^ ^41^ ^]^ methyl formate^[^ ^42^ ^]^ and dimethyl acetal^[^ ^43^ ^]^) were obtained as by‐products, greatly decreasing the efficiency of methanol steam reforming.^[^ ^44^ ^]^ So this reaction, which is typically catalyzed by heterogeneous catalysts, often requires high pressure and high temperature (200–300 °C).^[^ ^45^ ^]^ However, CO can be easily generated and poison the catalytic system of fuel cell, as well as contaminate the H_2_ gas under such high temperature.^[^ ^46^, ^47^ ^]^ Therefore, it is highly desirable to develop the efficient catalytic systems for H_2_ production from methanol at much lower temperature.^[^ ^48^, ^49^, ^50^ ^]^ For example, Zhou's group first reported [Cp*IrCl(phen)]Cl catalyzed H_2_ generation from methanol at near‐room temperature.^[^ ^51^ ^]^ Herein, we have first reported carbon nanotube‐supported Pt, Pd, and Rh nanocomposites (Pt/CNT, Pd/CNT, and Rh/CNT) as high‐performance nanocatalysts, via stabilization of Pt, Pd, and Rh nanoparticles onto carbon nanotube (CNT),^[^ ^52^, ^53^, ^54^ ^]^ for H_2_ production upon methanol dehydrogenation at 30 °C (Equation 1). Therein, the optimal Pt/CNT nanocomposite presented the superior catalytic activity in H_2_ evolution upon methanol dehydrogenation at the expense of B_2_(OH)_4_, with a TOF value of 299.51 min^−1^ at 30 °C. Among them, B_2_(OH)_4_ was frequently applied in borylation reaction and reduction reaction,^[^ ^22^ ^]^ and recently used as the sacrificial agent for H_2_ production.^[^ ^55^, ^56^ ^]^ In order to speculate and verify its mechanism, the carrier effect, kinetic study, kinetic isotope effect, tandem reaction, and density functional theory (DFT) of H_2_ production upon methanol dehydrogenation had been scrutinized in detail. Interestingly, H_2_ was also successfully produced from methanol dehydrogenation at −10 °C, which absolutely solved the freezing problem in the H_2_ evolution upon water‐splitting reaction.

(1) $$2\mathrm{MeOH}+B_{2}{\left(\mathrm{OH}\right)}_{4}\to 2\mathrm{MeOB}{\left(\mathrm{OH}\right)}_{2}+H_{2}↑$$

## 2 Results and Discussion

### 2.1 The Methanol Dehydrogenation Catalyzed by Different Nanocomposites

**Scheme 1:**
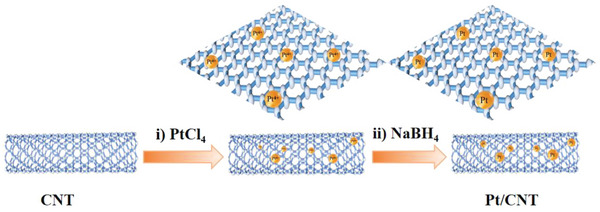
The synthesis of Pt/CNT.

**Figure 1:**
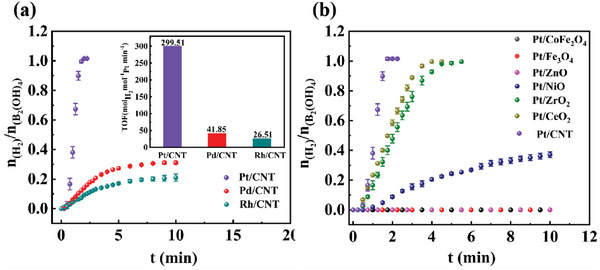
H_2_ evolution catalyzed by a) Pt/CNT, Pd/CNT, and Rh/CNT; b) Pt/CoFe_2_O_4_, Pt/Fe_3_O_4_, Pt/ZnO, Pt/NiO, Pt/ZrO_2_, Pt/CeO_2_, and Pt/CNT. Reaction condition: B_2_(OH)_4_ (2 mmol), catalyst (0.2 mol.%), and MeOH (2 mL).

As demonstrated in **Scheme** **1**, Pt/CNT, Pd/CNT, and Rh/CNT were prepared by using metal salts (including PtCl_4_, K_2_PdCl_4,_ and Rh(NO_3_)_3_) and CNT, followed by NaBH_4_ reduction in H_2_O at 30 ^o^C, respectively. Then catalytic activities of Pt/CNT, Pd/CNT, and Rh/CNT for H_2_ production upon methanol dehydrogenation at the expense of B_2_(OH)_4_ were recorded in **Figure** **1**a. The result exhibited that Pt/CNT showed a much higher TOF (299.51 min^−1^) than those of Pd/CNT (41.85 min^−1^) and Rh/CNT (26.52 min^−1^) in H_2_ production upon methanol dehydrogenation. Next, some common carriers, including CeO_2_, ZrO_2_, NiO, ZnO, Fe_3_O_4_, and CoFe_2_O_4_, had also been measured for methanol dehydrogenation. Pt/CeO_2_ (Figure S1, Supporting Information), Pt/ZrO_2_ (Figure S2, Supporting Information), Pt/NiO (Figure S3, Supporting Information), Pt/ZnO (Figure S4, Supporting Information), Pt/Fe_3_O_4_ (Figure S5, Supporting Information) and Pt/CoFe_2_O_4_ (Figure S6, Supporting Information) nanocomposites were obtained at standard condition. As displayed in Figure 1b, the order of TOF value in H_2_ production follows: Pt/CNT (299.51 min^−1^) > Pt/CeO_2_ (200.28 min^−1^) > Pt/ZrO_2_ (133.17 min^−1^) > Pt/NiO (27.90 min^−1^), whereas Pt/ZnO, Pt/Fe_3_O_4_ and Pt/CoFe_2_O_4_ were catalytically inactive (Figure S7, Supporting Information). In summary, Pt/CNT exhibited the superior TOF value of 299.51 min^−1^ in H_2_ production, emphasizing the critical role of CNT in methanol dehydrogenation. It seems that the confinement of Pt nanoparticles by CNTs is conducive to inhibiting the aggregation of Pt nanoparticles, thereby significantly increasing its catalytic performance and stability.

### 2.2 Characterization of Pt/CNT

**Figure 2:**
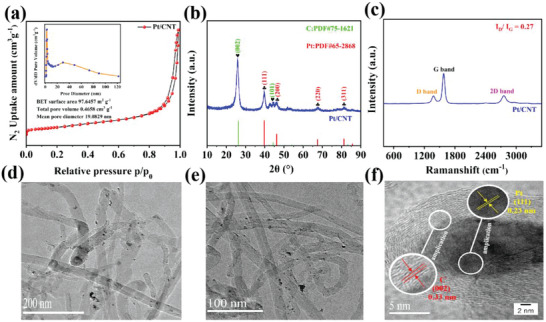
a) BET, b) XRD, c) Raman spectrum, d,e) TEM, and f) HRTEM of Pt/CNT.

**Figure 3:**
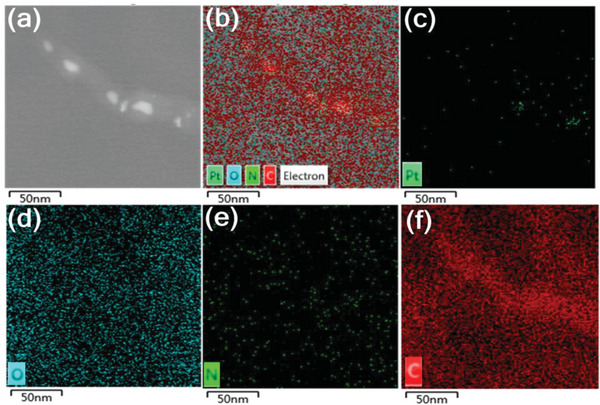
a) STEM, b) combined Pt, O, N, and C, c) Pt, d) O, e) N, and f) C EDX mapping of Pt/CNT.

As illustrated in **Figure** **2**a, the BET result presented the BET surface area, total pore volume and mean pore diameter of Pt/CNT nanocomposite is 97.64 m^2^ g^−1^, 0.47 cm^3^ g^−1^, and 19.08 nm, respectively, indicating that Pt/CNT possessed a mesoporous structure.^[^ ^57^ ^]^ A distinct characteristic peak at 26^o^, which is corresponding to graphene (002) (JCPDS card No. 75‐1621), was recorded in Figure 2b.^[^ ^58^ ^]^ The distinct peaks of Pt (311), Pt (220), Pt (200), and Pt (111) at 81.5^o^, 68.1^o^, 46.7^o^ *resp*. 39.8^o^ were recorded in XRD, indicating that PtNPs were stabilized onto CNT (JCPDS card No. 65‐2868).^[^ ^59^ ^]^ Next, the Pt content of Pt/CNT was measured by ICP to be 4.46 wt.%, which is just slightly lower than the theoretical value (4.65 wt.%). The obvious peaks of D‐band (1383.02 cm^−1^), G‐band (1584.86 cm^−1^) and 2D‐band (2758.13 cm^−1^) were observed in Figure 2c.^[^ ^60^ ^]^ Among them, the G‐band and D‐band were corresponding to graphite carbon *resp*. disordered carbon. The small value of *I*_D_/*I*_G_ (0.27) illustrated that there were only few crystal defects in Pt/CNT. The external nano‐structure and nano‐morphology Pt/CNT nanocomposite had also been measured by TEM and HRTEM. As illustrated in Figure 2d,e, Pt/CNT nanocomposite possessed a nanotube structure. Some Pt nanoparticles (3.79 nm, Figure S8, Supporting Information) were located at the surface of CNT, other Pt nanoparticles were encapsulated into CNT. C (002), where its crystal lattice spacing is 0.33 nm, was recorded in the exterior (Figure 2f). While Pt (111) of 0.23 nm was presented at the interior, further confirming some Pt nanoparticles were encapsulated into CNT.^[^ ^61^ ^]^ Moreover, the precise localization of C, N, O, and Pt elements in Pt/CNT nanocomposite had been further characterized by EDX. As shown in **Figure** **3**, the surface of Pt/CNT was consisted of Pt, O, N, and C elements. It is obvious that some Pt nanoparticles were confined into CNT. This confinement of Pt nanoparticles by CNTs is conducive to inhibiting the aggregation of Pt nanoparticles, thereby significantly increasing its catalytic performance and stability.^[^ ^62^, ^63^, ^64^ ^]^

**Figure 4:**
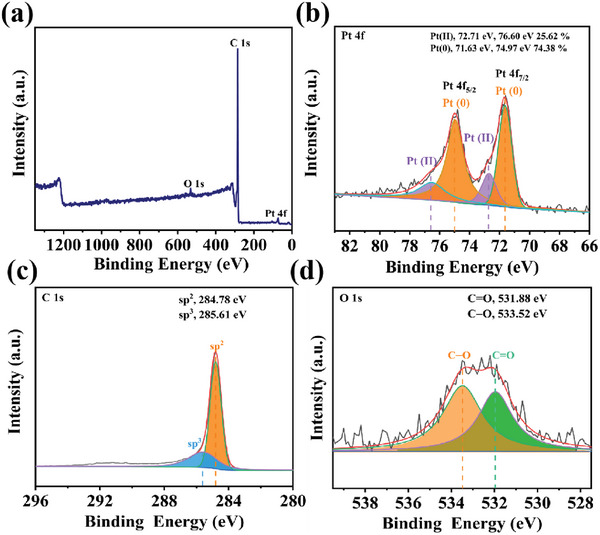
a) sum spectrum, b) Pt 4f, c) C 1s, and d) O 1s XPS of Pt/CNT.

In addition, the chemical valence states of surface elements of Pt/CNT had also been determined by XPS in **Figure** **4**a. The high‐resolution Pt 4f_7/2_ spectrum of Pt/CNT was divided into two peaks at 71.63 and 72.71 eV, which were attributed to Pt (0) (74.38%) *resp*. Pt (II) (25.63%) species (Figure 4b).^[^ ^65^ ^]^ This result had indicated that Pt (0) was partly oxidized to Pt (II) by O_2_. As illustrated in Figure 4c, the C 1s spectrum was decomposed into three characteristic peaks of C sp^2^ at 284.78 eV and C sp^3^ at 285.61 eV, respectively.^[^ ^66^ ^]^ As shown in Figure 4d, the O 1s spectrum of Pt/CNT nanocomposite was divided into two typical peaks of 531.88 and 533.52 eV, assigned to C═O and C─O, respectively.^[^ ^67^ ^]^ These O‐containing functional group in CNTs could protect the Pt nanoparticles by enhancing its stability and decreasing the leaching of Pt in the confined space. In addition, the XPS of Pt/ZrO_2_ was also measured in Figure S9 (Supporting Information), a slight shift was found in Pt 4f of Pt/CNT as compared to Pt 4f of Pt/ZrO_2_, suggesting the electron transfer from Pt atom into CNT surface. Thus, the superior catalytic performance of Pt/CNT in methanol dehydrogenation might be ascribed to its electronic interaction effect and synergistic effect.^[^ ^68^ ^]^

**Figure 5:**
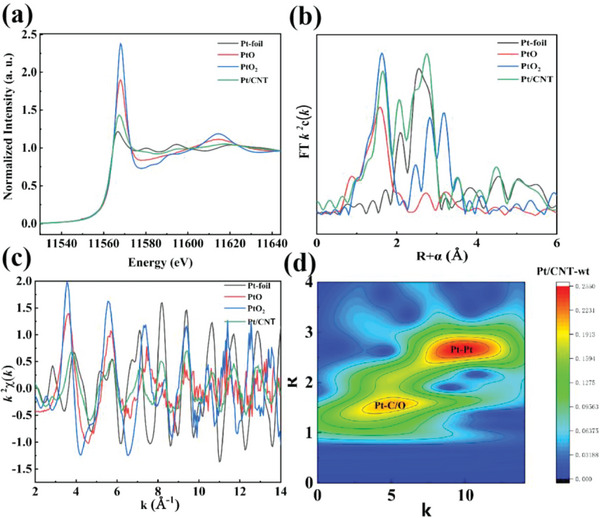
a) XANES spectra of the Pt L3‐edge over the Pt/CNT catalyst, Pt foil, PtO, and PtO_2_; b) FT‐EXAFS spectra of Pt/CNT catalyst, Pt foil, PtO, and PtO_2_; c) EXAFS k space fitting curves of Pt/CNT catalyst, Pt foil, PtO, and PtO_2_; d) Wavelet transformed XAS signal of Pt/CNT.

In order to further identify the coordination and valence states of Pt/CNT, X‐ray absorption near edge structure (XANES) spectra of the Pt L3‐edge over Pt/CNT catalyst, Pt foil, PtO, and PtO_2_ had been performed in **Figure** **5**a. the green peak of Pt/CNT was lower than that of PtO and slightly higher than that of Pt foil reference, suggesting the chemical charge of Pt atoms in Pt/CNT is between 0 and +2.^[^ ^69^ ^]^ Then, the Fourier transform extended X‐ray absorption fine structure (FT‐EXAFS) spectra of Pt/CNT uncovered that the presence of Pt–Pt, Pt–O–Pt, and Pt–C/O coordinations (Figure 5b,c),^[^ ^70^, ^71^ ^]^ demonstrating that Pt nanoparticles had been successfully stabilized by CNT. As shown in Figure 5d, the presence of Pt–Pt and Pt–C/O coordinations was also confirmed by wavelet transformed XAS analysis.^[^ ^72^ ^]^ In Table S1 (Supporting Information), the coordination configurations of Pt atoms in Pt/CNT were measured by quantitative least‐squares EXAFS best‐fitting analysis. The coordination number of Pt–C/O, Pt–Pt, and Pt–O–Pt was 1.6, 4.8, and 2.0, respectively. In summary, these results further confirmed that chemical charge of Pt atoms in Pt/CNT is between 0 and +2, which was in line with XPS.

### 2.3 Kinetic Study

**Figure 6:**
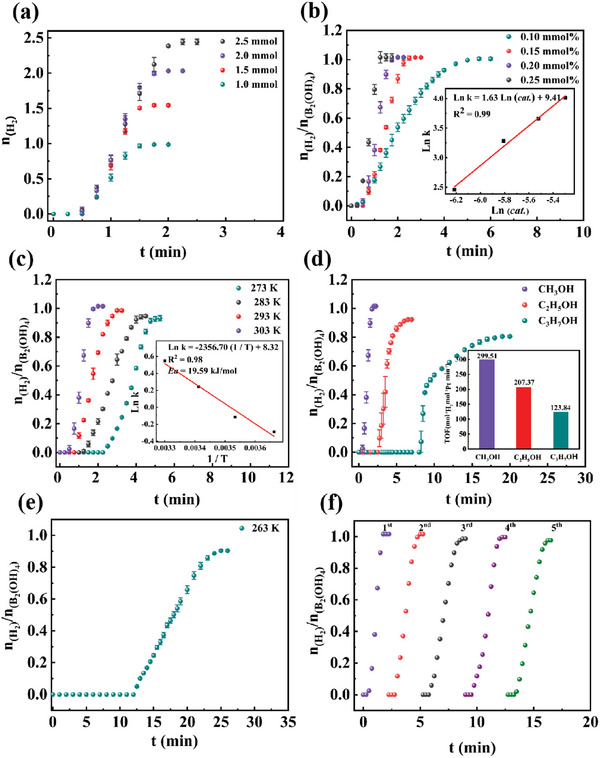
Plots of obtained H_2_ volume versus time for H_2_ evolution from MeOH with a) different concentrations of B_2_(OH)_4_, b) various amounts of Pt/CNT, c) various reaction temperatures; d) CH_3_OH, C_2_H_5_OH and C_3_H_7_OH, e) at 263 K, f) Stability test on the Pt/CNT catalyst in H_2_ evolution.

The kinetic study (such as B_2_(OH)_4_ dosage, Pt/CNT amount, and reaction temperature) of H_2_ production upon methanol dehydrogenation at the expense of B_2_(OH)_4_ was further studied in detail. First, the H_2_ production catalyzed by 0.2 mol.% Pt/CNT was conducted in the various B_2_(OH)_4_ amounts from 1.0 to 2.5 mmol at MeOH (2 mL). In **Figure** **6**a, the H_2_ production rate was independent of B_2_(OH)_4_ amounts, suggesting H_2_ production was a zero‐order reaction in B_2_(OH)_4_ concentration. In general, 1 mol of H_2_ was generated upon the expense of 1 mol of B_2_(OH)_4_. While the H_2_ production rate boosted with the increment of Pt/CNT concentration (Figure 6b), with the slope was 1.67, indicating H_2_ production was a first‐order reaction in Pt/CNT concentration. In order to obtain *E*_a_ of H_2_ production upon methanol dehydrogenation over Pt/CNT, the methanol dehydrogenation was conducted from 273 to 303 K (Figure 6c). Based on the Arrhenius law, the *E*_a_ was found to 19.59 kJ mol^−1^. Then other common alcohols, including ethanol and propanol, had also been tested for H_2_ production. As described in Figure 6d, H_2_ was also successfully generated from ethanol (207.37 min^−1^) and propanol (123.84 min^−1^), but it needed 2.5 and 8 min induction time, respectively. Interestingly, H_2_ was also successfully produced from methanol dehydrogenation at −10 °C (Figure 6e), which absolutely solved the freezing problem in the H_2_ evolution upon water‐splitting reaction.

### 2.4 Stability of Pt/CNT Nanocomposite

It is also vital to demonstrate the stability of Pt/CNT in H_2_ production upon methanol dehydrogenation at the expense of B_2_(OH)_4_ for the further industrial and practical application. As described in Figure 6f, the result confirmed that Pt/CNT still kept excellent H_2_ production rate after at least five runs in methanol dehydrogenation. Then, the 5^th^ recycled Pt/CNT nanocomposite was further measured by TEM and XPS. Figure S10 (Supporting Information) exhibited the size of 5^th^ recycled Pt/CNT nanocomposite (3.92 nm, Figure S11, Supporting Information) kept the same as fresh one (3.79 nm). In Figure S12 (Supporting Information), the contents of Pt (II) and Pt (0) of 5^th^ recycled Pt/CNT also remained the same as the fresh one. Indeed, H_2_ production rate remained unchanged after five times recycling of Pt/CNT, suggesting that Pt/CNT nanocomposite was an excellent stable, heterogeneous, and recyclable nanocatalyst for methanol dehydrogenation. Moreover, the commercial Pt/C was also tested for H_2_ production upon methanol dehydrogenation. Although commercial Pt/C was as efficient as Pt/CNT in H_2_ production upon methanol dehydrogenation at first, the catalytic activity of Pt/C greatly deceased after five runs in methanol dehydrogenation Figure S13 (Supporting Information). According to TEM pictures, we found the size of 5^th^ recycled Pt/C had increased from 3.77 (Figures S14,S15, Supporting Information) to 5.15 nm (Figures S16,S17, Supporting Information). More importantly, H_2_ production rate still remained unchanged after ten times recycling of Pt/CNT (Figure S18, Supporting Information). In summary, the confinement of Pt nanoparticles by CNTs is conducive to inhibiting the aggregation of Pt nanoparticles, thereby significantly increasing its catalytic performance and stability.

### 2.5 The Mechanism of Methanol Dehydrogenation

**Figure 7:**
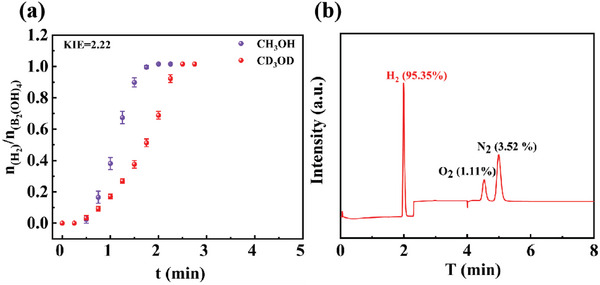
a) H_2_ evolution from MeOH catalyzed by Pt/CNT in CD_3_OD (red) or CH_3_OH (blue); b) GC spectra of evoluted gas mixture from MeOH over Pt/CNT at 30 °C.

H_2_ production upon methanol dehydrogenation was performed in CH_3_OH *resp*. CD_3_OD was recorded in **Figure** **7**a. A large KIE of 2.22 was obtained indicating that the breaking of O─H bond of MeOH is the rate‐controlling step for methanol dehydrogenation.^[^ ^73^ ^]^ In Figure 7b, the gas mixture obtained from methanol dehydrogenation was confirmed by gas chromatograms (GC) to be only H_2_, illustrating that H_2_ generation upon methanol dehydrogenation over Pt/CNT has been successfully realized towards fuel cell power systems.

**Figure 8:**
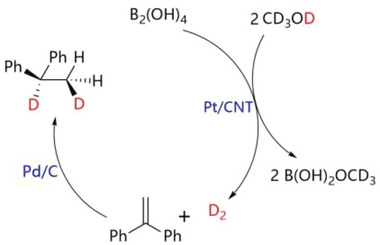
Tandem reaction for 1,1‐diphenylethylene hydrogenation using D_2._

H_2_ generation upon methanol dehydrogenation is not only applied in the safe and efficient generation, storage, and transportation of H_2_, but also in its in situ tandem reactions. As shown in Figure S19 (Supporting Information), tandem reaction was carried out in a dual‐chamber reactor for 1,1‐diphenylethylene hydrogenation with in situ produced H_2_ from methanol dehydrogenation, and the target product of 1,1‐diphenylethane was provided in > 99% yield, which was confirmed by ^1^H‐NMR (Figure S20, Supporting Information). In addition, the by‐production of B(OH)_2_OMe was further identified by mass spectrum (Figure S21, Supporting Information) and ^1^H‐NMR (Figure S22, Supporting Information). As shown in **Figure** **8**, D_2_ was successfully generated from CD_3_OD dehydrogenation, then deuterated 1,1‐diphenylethane was also obtained in > 99% yield, which was confirmed by ^1^H‐NMR (Figure S23, Supporting Information). The formation of Ph_2_CD‐CH_2_D was also verified by mass spectrum (Figure S24, Supporting Information). These result suggested both H atoms of H_2_ are supplied by CH_3_OH.

### 2.6 DFT Calculation

**Figure 9:**
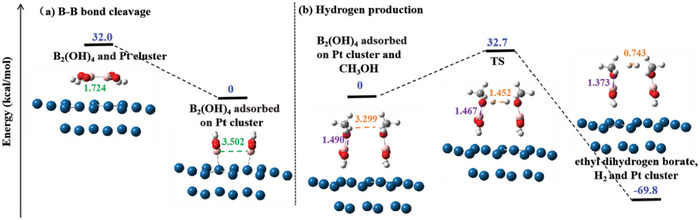
Relative electronic energy (blue) diagram for H_2_ generation over Pt/CNT.

To further verify the mechanism of H_2_ generation upon methanol dehydrogenation, the systematic DFT calculation was also performed. According to our previous work,^[^ ^74^ ^]^ Pt_18_ clusters and methanol were chosen as models for H_2_ generation upon methanol dehydrogenation, the plausible mechanism pathway and energy change of H_2_ generation upon methanol dehydrogenation were concluded in **Figure** **9**. First, B_2_(OH)_4_ was adsorbed on Pt_18_ clusters to give **I**, the bond length of B‐B bond was enhanced from 1.724 to 3.502 Å (green line), indicating B─B bond was completely disconnected. 32 kcal mol^−1^ energy was released, suggesting that this reaction was spontaneous. Subsequently, H_2_ and MeOB(OH)_2_ were formed, via TS (II) (H─H bond length and B─H bond length are 1.372 Å *resp*. 1.451 Å), by the reaction of B_2_(OH)_4_ and methanol at the surface of Pt_18_ cluster (H─H bond length and B─H bond length are 2.893 Å *resp*. 1.496 Å). The gradual shortening of the H─H (orange dotted line) and B─H (purple dotted line) bond lengths had proved the formation of H_2_ and MeOB(OH)_2_. The activation energy (∆E1) and energy difference (∆E2) were 32.7 and −69.8 kcal mol^−1^, respectively, suggesting our speculative mechanism is feasible.

**Figure 10:**
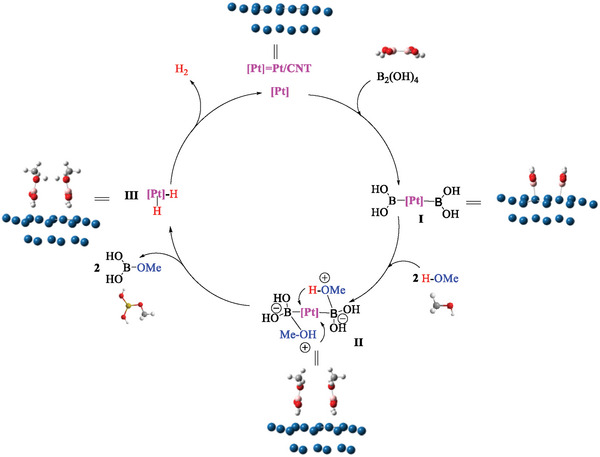
The proposed mechanism of H_2_ generation upon methanol dehydrogenation.

Based on our control experiments, tandem reaction and DFT calculation, the feasible mechanism of methanol dehydrogenation was proposed in **Figure** **10**. First, B_2_(OH)_4_ reacted with Pt/CNT to give intermediate **I**, being subsequently converted into intermediate **II** by the attack of MeOH molecules. The large KIE of 2.22 indicated that the breaking of O─H bond of MeOH was the rate‐controlling step for methanol dehydrogenation. Finally, Pt(H)_2_ species **III** was generated from intermediate **II** by releasing MeOB(OH)_2_, simultaneously providing H_2_.

## 3 Conclusion

In summary, a sequence of carbon nanotube‐supported Pt, Pd, and Rh nanocomposites (Pt/CNT, Pd/CNT, and Rh/CNT) have been designed and synthesized as high‐performance nano‐catalysts, via stabilization of Pt, Pd, and Rh nanoparticles onto CNT, for H_2_ production upon methanol dehydrogenation. Therein, the optimal Pt/CNT presented the superior catalytic activity in H_2_ production upon methanol dehydrogenation at the expense of B_2_(OH)_4_, with a TOF value of 299.51 min^−1^ at 30 °C. Compared with other common carriers, including CeO_2_, ZrO_2_, NiO, ZnO, Fe_3_O_4_, and CoFe_2_O_4_, Pt/CNT exhibited the superior catalytic performance in H_2_ evolution, emphasizing the critical role of CNT in methanol dehydrogenation. The confinement of Pt nanoparticles by CNTs is conducive to inhibiting the aggregation of Pt nanoparticles, thereby significantly increasing its catalytic performance and stability. The kinetic study (including Pt/CNT concentration, B_2_(OH)_4_ amount, and dehydrogenation temperature) and detailed mechanistic insights, particularly KIE test, tandem reaction, GC result, and DFT calculation, confirmed that the breaking of O─H bond of MeOH was the rate‐controlling step for methanol dehydrogenation, and both H atoms of H_2_ were supplied by CH_3_OH. Interestingly, H_2_ was also successfully produced from methanol dehydrogenation at −10 °C, which absolutely solved the freezing problem in the H_2_ evolution upon water‐splitting reaction.

A drawback of H_2_ production upon methanol dehydrogenation is the difficulty of regenerating B_2_(OH)_4_ from MeOB(OH)_2_, but this challenge is also appropriate for the other hydroborons.^[^ ^75^ ^]^ Further study about the regeneration of B_2_(OH)_4_ from MeOB(OH)_2_ is currently under investigation in our group.

This work not only develops an efficient catalytic system for H_2_ production from methanol in sub‐zero temperatures, but it also proposes an easy and simple method for pure D_2_ production upon CD_3_OD dehydrogenation.

## Conflict of Interest

The authors declare no conflict of interest.

## Supporting information

Supporting Information

## Data Availability

The data that support the findings of this study are available from the corresponding author upon reasonable request.
