# Supplementary material for: Spatial Confinement of Pt Nanoparticles in Carbon Nanotubes for Efficient and Selective H2 Evolution from Methanol
Source: Adv Sci (Weinh). 2024 Jan 15;11(12):2306893. doi: 10.1002/advs.202306893 (PMC10966539; doi:10.1002/advs.202306893)
Supplement: Supplementary file 1 — Supporting Information [file ADVS-11-2306893-s001.pdf]

ADVANCED SCIENCE
Open Access

Supporting Information

for *Adv. Sci.*, DOI 10.1002/advs.202306893

Spatial Confinement of Pt Nanoparticles in Carbon Nanotubes for Efficient and Selective H_2 Evolution from Methanol

Xiaotao Jin, Jiaying Yan, Xiang Liu, Qing Zhang, Yingping Huang, Yanlan Wang, Changlong Wang* and Yufeng Wu**

Supporting Information

Spatial confinement of Pt nanoparticles in carbon nanotubes for efficient and selective H_2 evolution from methanol

Xiaotao Jin,[a,1] Jiaying Yan,[a,1] Xiang Liu,[a,1]* Qing Zhang,[a] Yingping Huang,[a] Yanlan Wang,[a,c]

Changlong Wang[a,b]* and Yufeng Wu[a,b]*

[a]Engineering Research Center of Eco-environment in Three Gorges Reservoir Region, College of Materials and Chemical Engineering, China Three Gorges University, Yichang, Hubei 443002, P. R. China, E-mails: xiang.liu@ctgu.edu.cn (X. Liu)

[b]Institute of Circular Economy, Faculty of Materials and Manufacturing, Beijing University of Technology, Beijing 100124, China. E-mail: clwang1987@126.com (C. Wang) and wuyufeng3r@126.com (Y. Wu)

[c]Department of chemistry and chemical engineering, Liaocheng University, 252059 Liaocheng, China

[1]These authors have equally contributed to this work and should be considered as co-first authors

Table of Content

1. Chemicals and reagents S2

2. Characterization S2

3. Synthesis of Pt/CNT S2

4. H_2 evolution upon methanol dehydrogenation S3

5. Computational details and models S4

6. Table S1 .... S5

7. Characterization of catalysts (Fig. S1-S24) S5

8. Vibrational frequencies in all calculation S17

1. Chemicals and reagents

The chemicals are used without further purification, unless indicated. Ethanol (C_2H_5OH) was purchased from Hubei Shentry Chemical Technology Co., Ltd. Potassium tetrachloride palladium (K_2PdCl_4) and Carbon nanotubes (CNT) were purchased from Bide Pharmatech Co., Ltd. Zinc oxide (ZnO), Nickel oxide (NiO), Ferrosoferric oxide (Fe_3O_4) and Platinum chloride ($PtCl_4$) were purchased from Aladdin Reagent Co., Ltd. $NaBH_4$ was obtained from Shanghai Lingfeng Chemical Reagent Co., Ltd. Methanol-D_4 (CD_3OD), $Rh(NO_3)_3$, CeO_2, ZrO_2 were purchased from Shanghai Macklin Biochemical Co., Ltd. Methanol (CH_3OH) was purchased from Tianjin Fuyu Fine Chemical Co., Ltd. 1-Propanol (C_3H_7OH) was Anaiji (Shanghai) Pharmaceutical Chemistry Co., Ltd. Cobalt iron oxide ($CoFe_2O_4$) was obtained from Shanghai Yi En Chemical Technology Co., Ltd.

2. Characterization

Transmission electron microscopy (TEM) was measured by a JEOL-JEM. X-ray diffraction (XRD) analyses were performed on a D8 Advanced. X-ray photoelectron spectrometry (XPS) was performed on a Thermo SCIENTIFIC ESCALAB 250Xi. GC spectra of generated gas (H_2, CO_2) were detected using Agilent 7890B with thermal conductivity detector (TCD) and flame ionization detector (FID)-Methanator. The element contents of samples were determined by ICP-AES (Agilent 7700/7800 (MS), USA). Raman spectra of catalysts were measured on a Horiba scientific-LabRAM HR evolution. The Brunner-Emmet-Teller (BET) specific area, pore volume and pore size of Pd/CNT was measured by a ASAP2020 PLUS HD88. The obtained XAFS data was processed in Athena (version 0.9.26) for background, pre-edge line and post-edge line calibrations. Then Fourier transformed fitting was carried out in Artemis (version 0.9.26). The k^2 weighting, k-range of 3~14.0 Å^{-1} and R range of 1 - 3 Å were used for the fitting of Pt foil; The k_2 weighting, k-range of 3~13 Å^{-1} and R range of 1 - 3 Å were used for the fitting of sample. The four parameters, coordination number, bond length, Debye-Waller factor and E0 shift (CN, R, ΔE_0) were fitted without anyone was fixed, the σ_2 was set.

3. Synthesis of Pt/CNT

In typical, 2.5 mL of $PtCl_4$ aqueous solution (0.01 M) and 100 mg of CNT and 15 mL of deionized H_2O were added into 50 mL of flask, and continue stirring the above mixture for 1 hour. Then, $NaBH_4$ solution (0.125 M, 2 mL) was injected to the mixture, and continue stirring the above mixture for 0.5 hour at r.t. Finally, the precipitant (Pt/CNT) was separated by filtering, and washed with three times of H_2O and three times of EtOH, and dried at 50 ^oC for 12 h.

4. H_2 production upon methanol dehydrogenation

Generally, 16.8 mg of Pt/CNT (0.2 mol% Pt) and 2 mmol of $B_2(OH)_4$ were added into a Schlenk flask (30 °C). This flask was linked to a water-filled glass column by a gas outlet (**Scheme S1**). Once 2 mL of MeOH was added into the flask, and recorded the time and corresponding volume of H_2 gas, simultaneously. The corresponding volume of H_2 gas was determined periodically by variation of H_2O's volume in glass column. A quantitative conversion of $B_2(OH)_4$ produced 1.0 equivalents of H_2, and occupied ca. 25.6 mL at atmospheric pressure. Prior to the reactions, the volumes were measured at atmospheric pressure and corrected for water vapor pressure at room temperature. Turnover frequency (TOF) is measured to estimate the efficiency of catalyst through the follow equation:

$$TOF = \frac{n_{H2}}{n_{Pd} \cdot t}$$

n(H_2) is molar of hydrogen, n(Pt) is total molar of Pt and t is corresponding reaction time, calculated at 10% conversion of $B_2(OH)_4$.

Cycle experiment: The Pt/CNT was collected by the simple filtration, washed by NaOH (0.5 M) and H_2O for next cycle. Reaction condition: $B_2(OH)_4$ (2 mmol), Pt/CNT (0.2 mol%) and MeOH (2 mL) at 30 ^oC

The active energy (E_a) is calculated through the Arrhenius equation:

$$lnk = lnA - E_a/RT$$

A is the pre-exponential factor (min^{-1}), k is reaction rate constant, R is 8.314J/(mol·K） and T is reaction temperature (K)

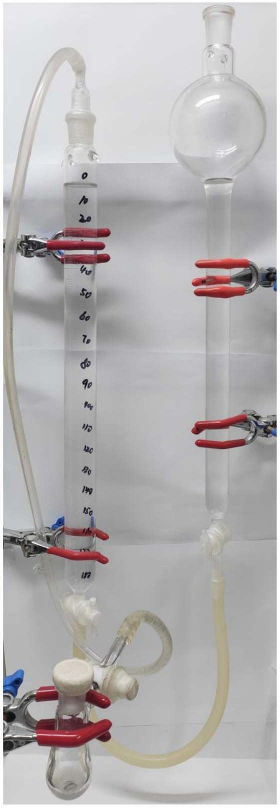

Scheme S1. The photo of the set-up for hydrogen generation

5. Computational details and models

Geometric structures of the reactant, products and transition states on the energy profiles are optimized by using B3LYP-D3 functional theory, with 6-31G+(d,p) basis set for C, B, O, H atoms and SDD for Pt atoms. Vibrational frequencies were done with harmonic approximation to make sure that all the geometric structures had no imaginary frequency and the transition state geometries had only one imaginary frequency. Transition state indeed connects the intended reactants and products by using intrinsic reaction coordinate (IRC) approach [*J. Phy. Chem.*, 1970, **74**, 4161-4163, *Acc. Chem. Res.*, 1981, **14**, 363-368]. The single point energy on the energy profiles are further calculated at B3LYP/def2tzvpp calculation level due to its higher accuracy. To simulate the effect of water environment, the polarizable continuum model (PCM) was selected.[Chem. Phys., 1981, 55, 117-129.] All calculations were conducted with Gaussian 16 program package. [Gaussian 16, Revision C. 01, Gaussian, Inc., *Wallingford CT*, **2016**] The energy barrier (activation energy, ΔE_1) is defined as the single point energy difference between the electronic energy of the transition state and that of the reactant. The single point energy difference between the energy of the product and that of the reactant is defined as the ΔE_2.

6. Table S1.

Table S1. EXAFS fitting parameters at the Pt K-edge for various samples（S_0^2=0.85）

	Shell	CN	R(Å)	σ^2	ΔE_0	R factor
Pt foil	Pt-Pt	12	2.76±0.01	0.0048	6.8±0.4	0.0024
	Pt-C/O	1.6±0.2	2.01±0.01	0.0043		
Pt/CNT	Pt-Pt	4.8±0.2	2.74±0.01	0.0054	4.4±1.3	0.0025
	Pt-O-Pt	2.0±0.5	3.04±0.01	0.0097		

[a]*CN*: coordination numbers; [b]*R*: bond distance; [c]σ^2: Debye-Waller factors; [d] ΔE_0: the inner potential correction. *R* factor: goodness of fit

7. Characterization of catalysts (Fig. S1-S24)

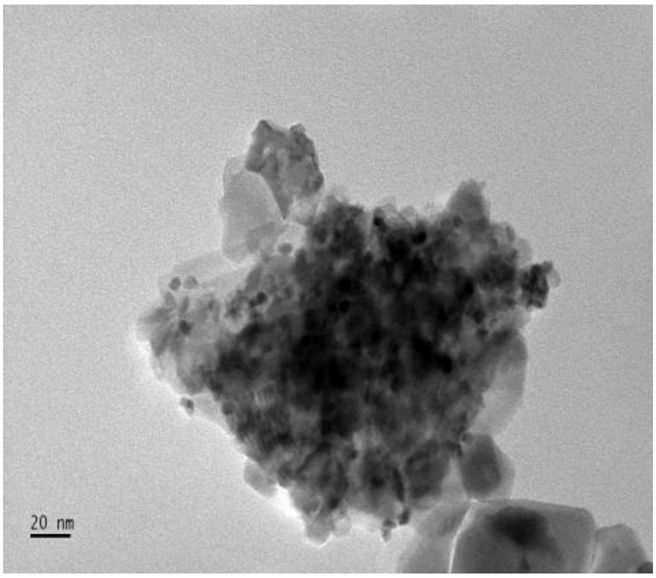

Figure S1. TEM of Pt/CeO_2

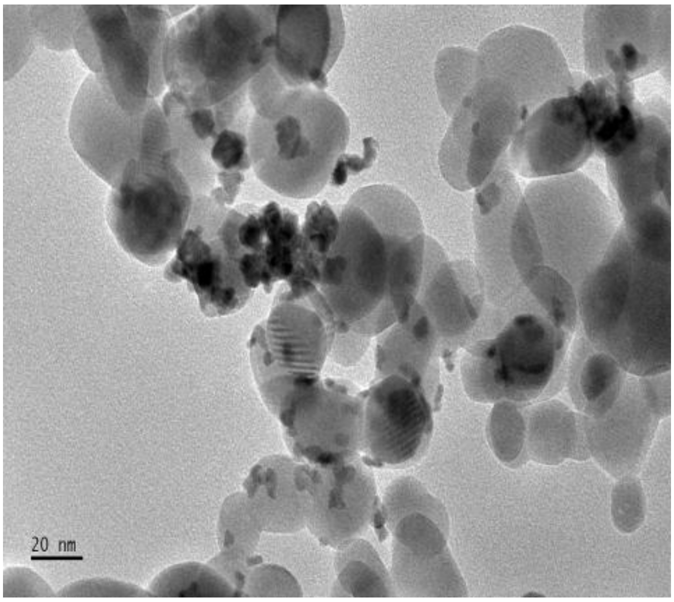

Figure S2. TEM of Pt/ZrO_2

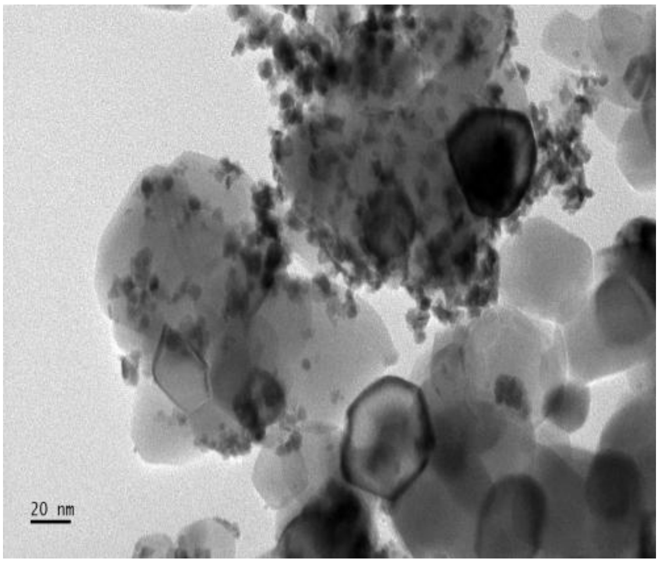

Figure S3. TEM of Pt/NiO

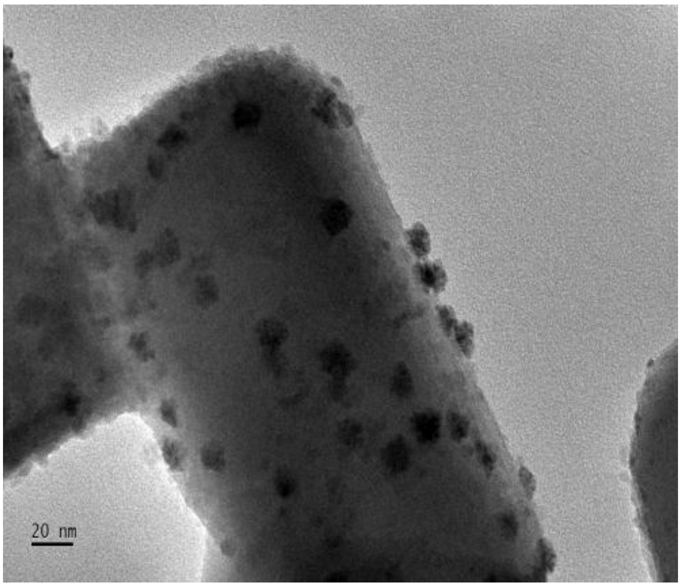

Figure S4. TEM of Pt/ZnO

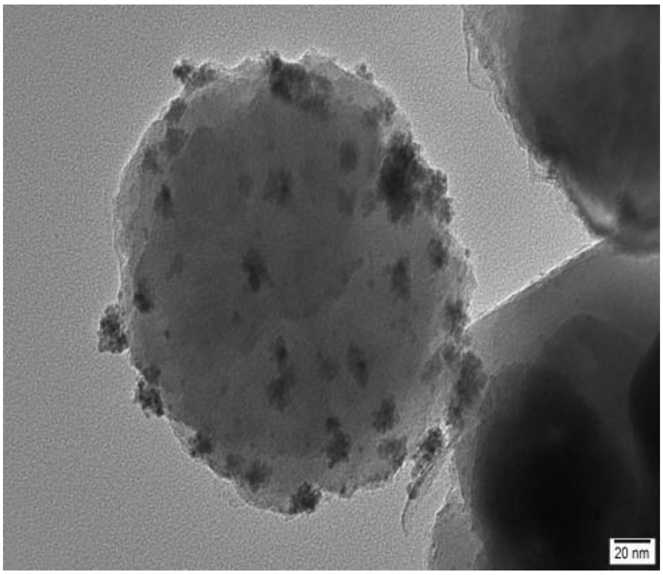

Figure S5. TEM of Pt/Fe_3O_4

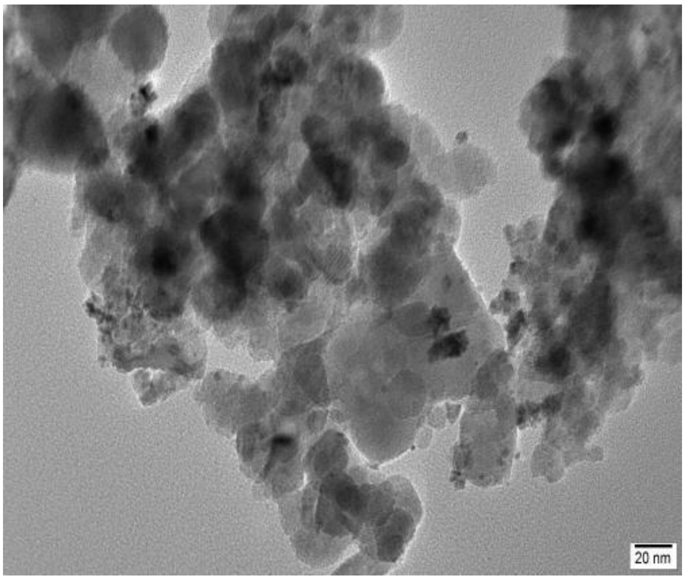

Figure S6. TEM of Pt/$CoFe_2O_4$

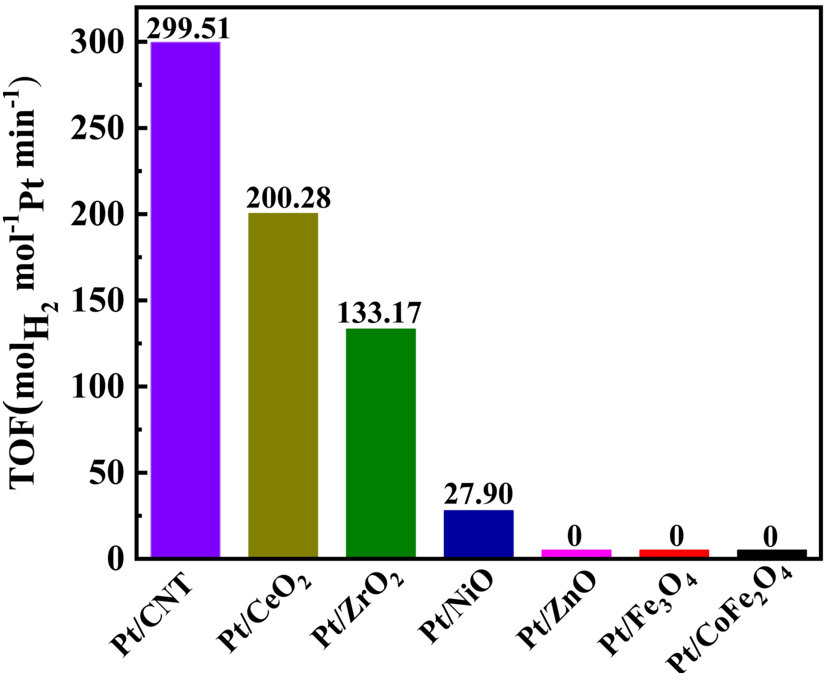

Figure S7. The corresponding TOF value.

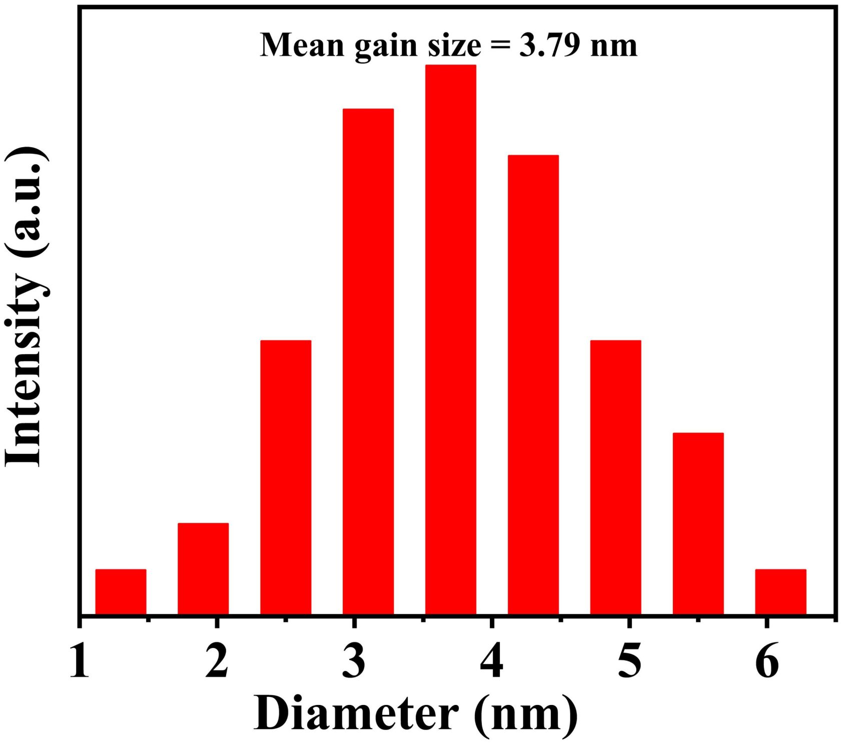

Figure S8. Distribution diagram of Pt/CNT

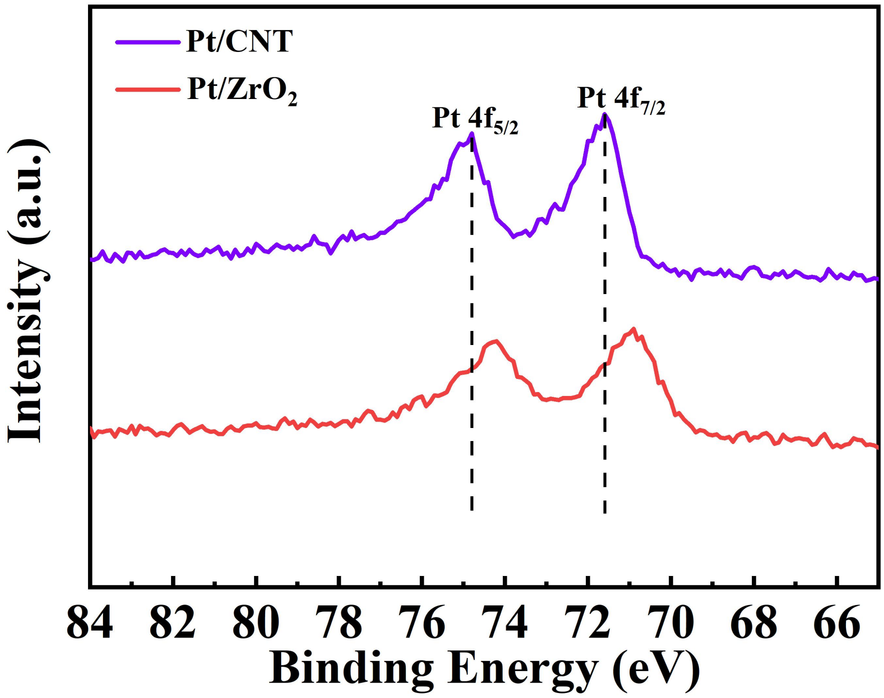

Figure S9. Pt 4f XPS of Pt/CNT and Pt/ZrO_2.

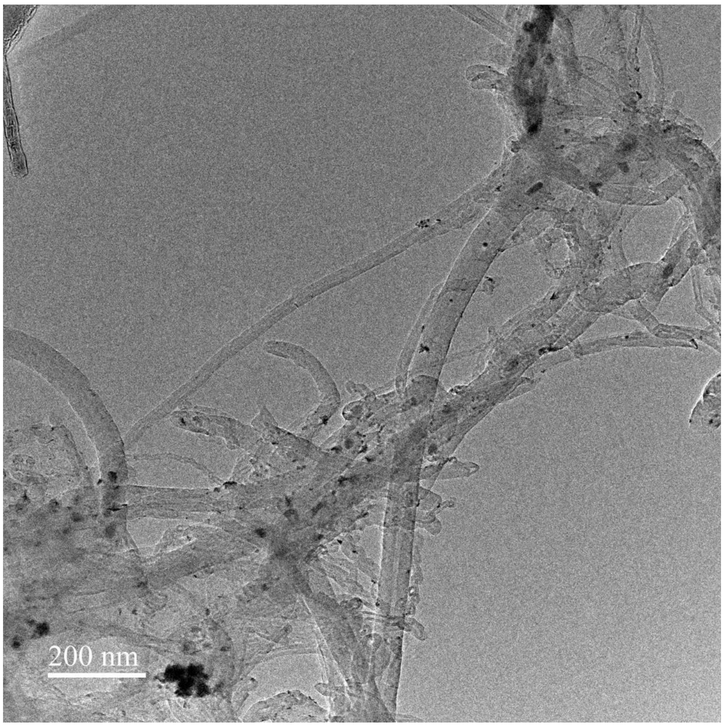

Figure S10. TEM of 5th reused Pt/CNT

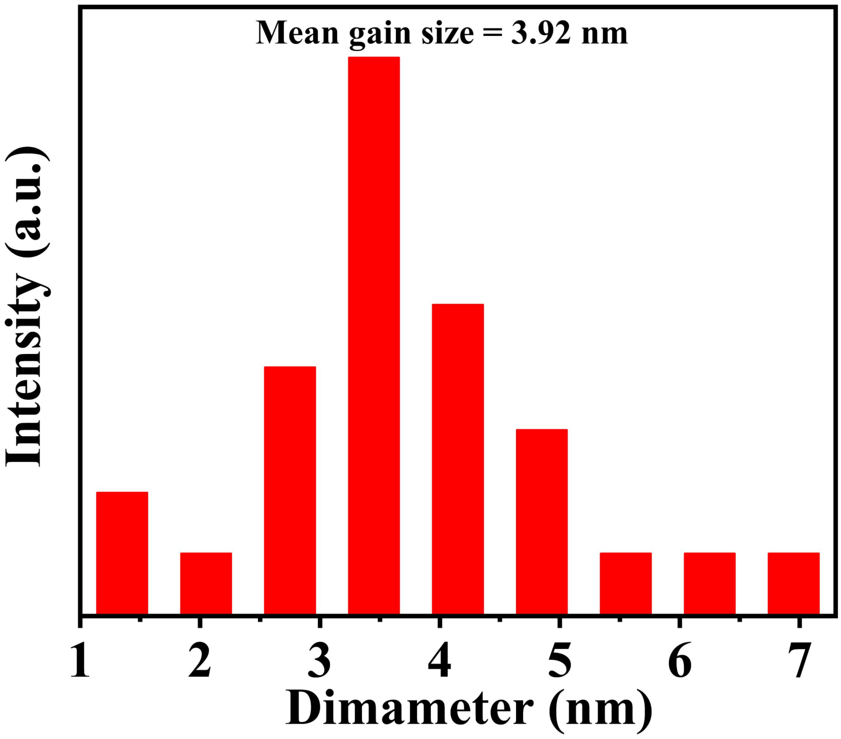

Figure S11. Distribution diagram of 5th reused Pt/CNT

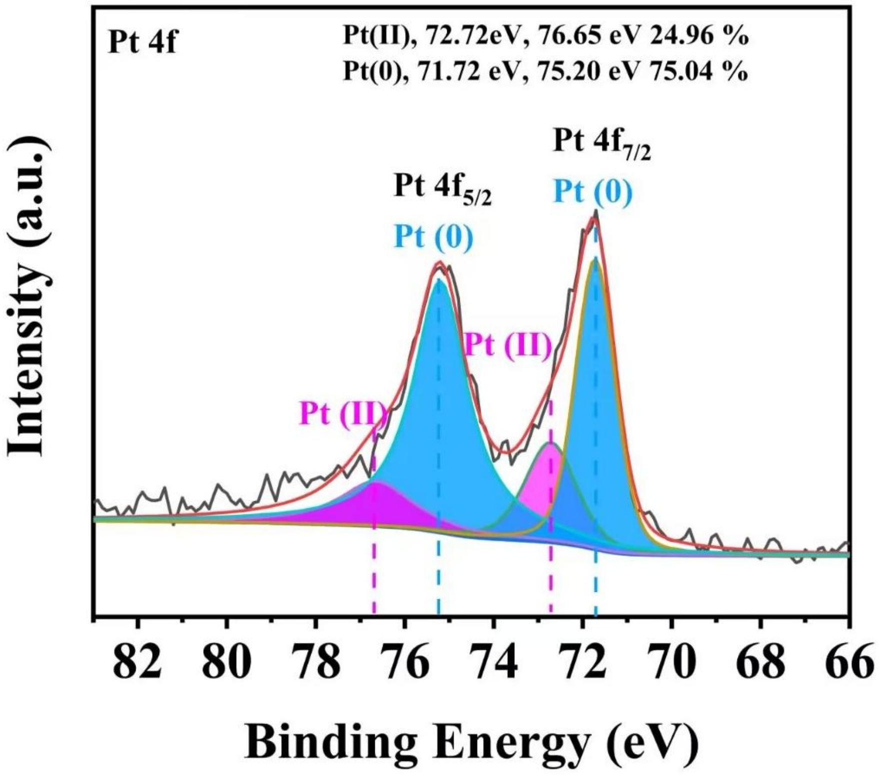

Figure S12. Pt 4f XPS of 5th reused Pt/CNT

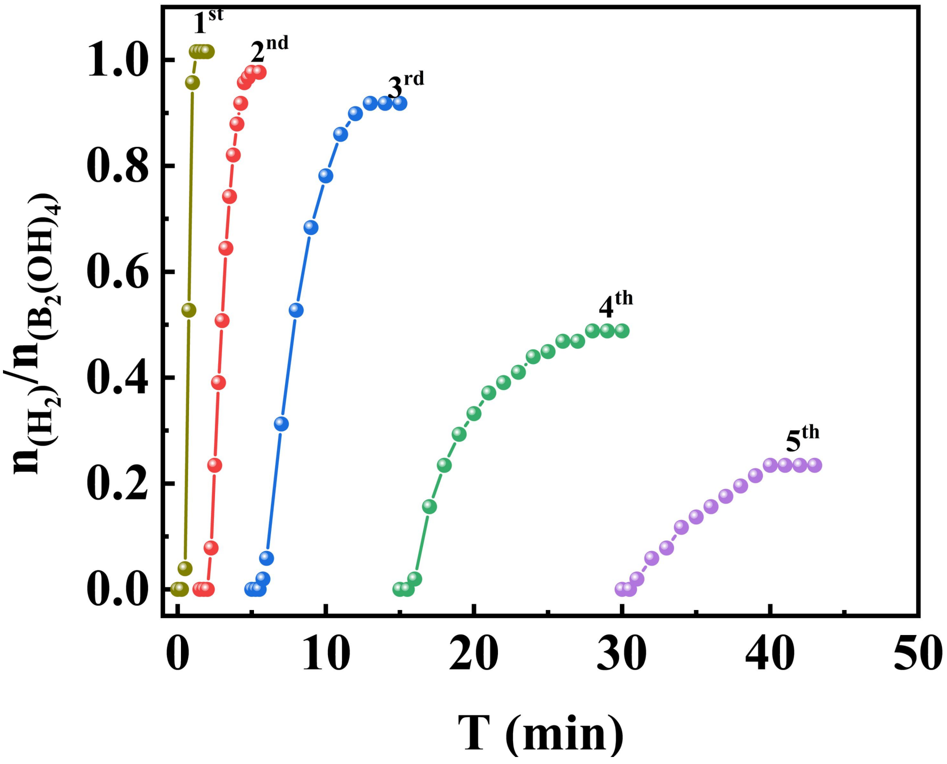

Figure S13. Stability test on the Pt/C catalyst in H_2 evolution

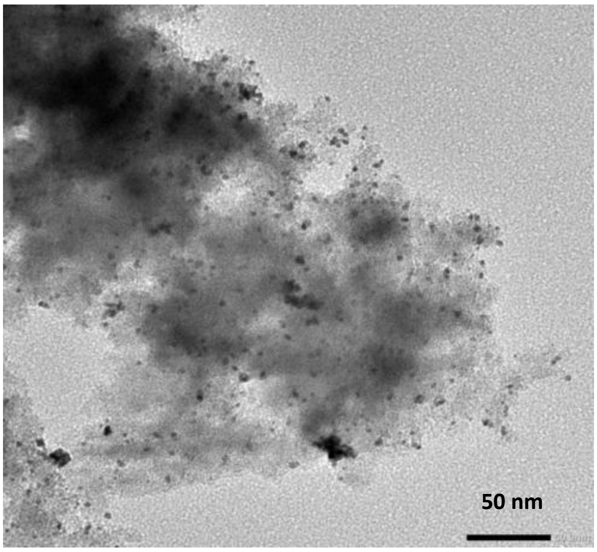

Figure S14. TEM image of fresh Pt/C.

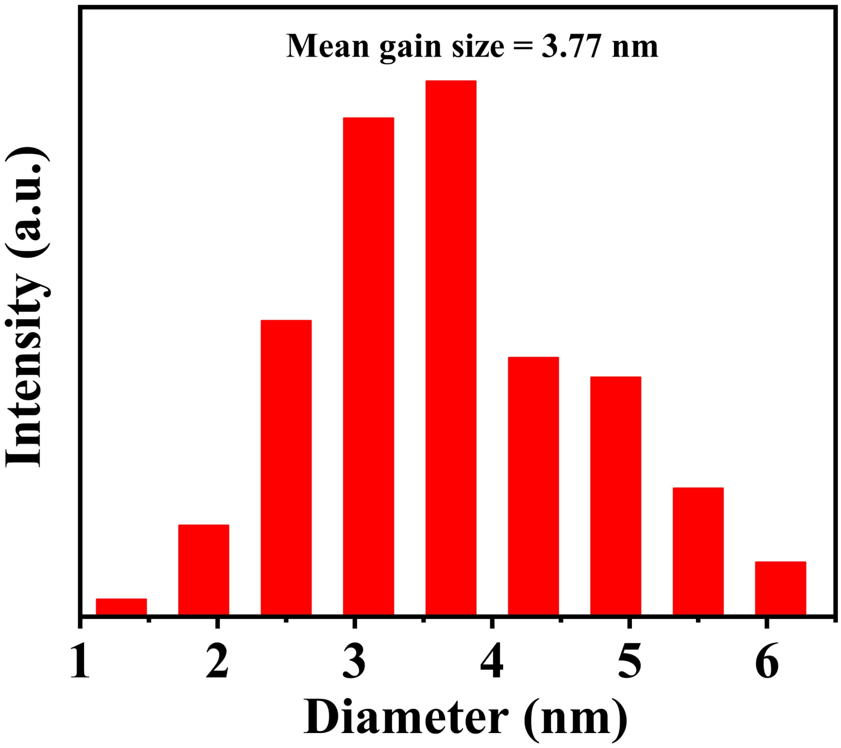

Figure S15. Distribution diagram of fresh Pt/C.

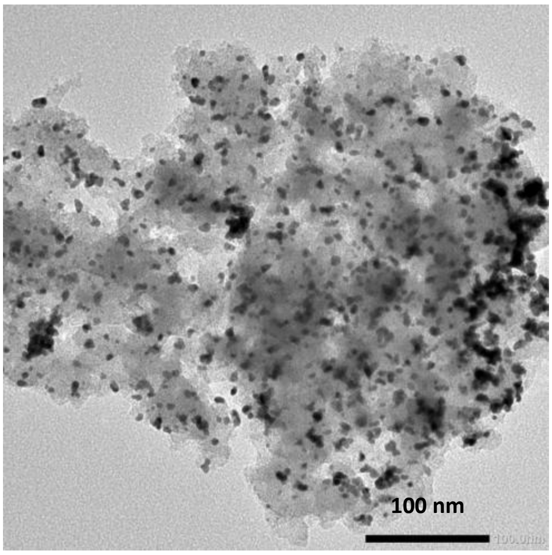

Figure S16. TEM image of 5th reused Pt/C.

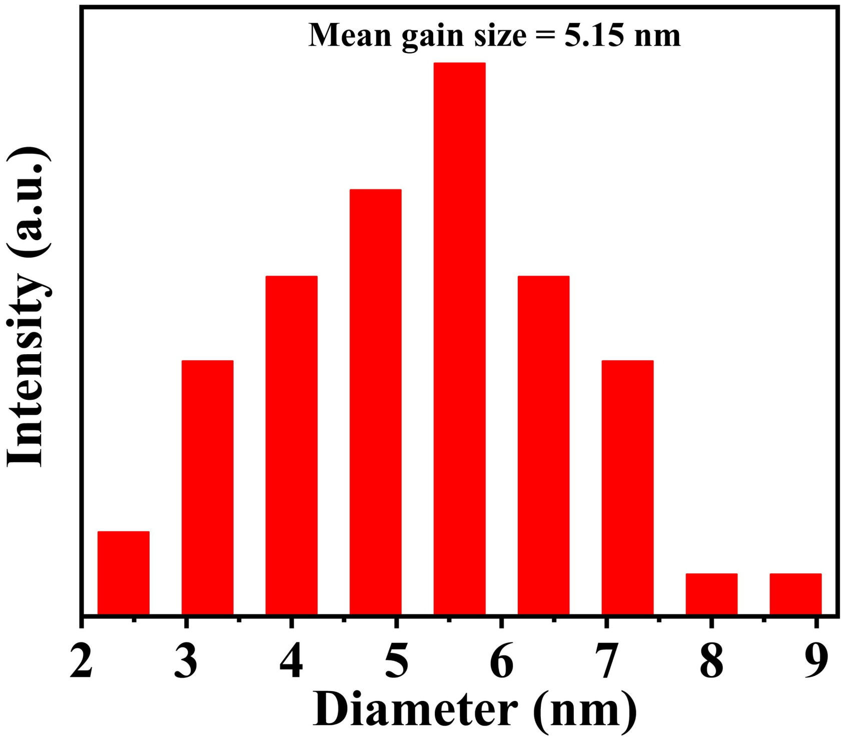

Figure S17. Distribution diagram of 5th reused Pt/C.

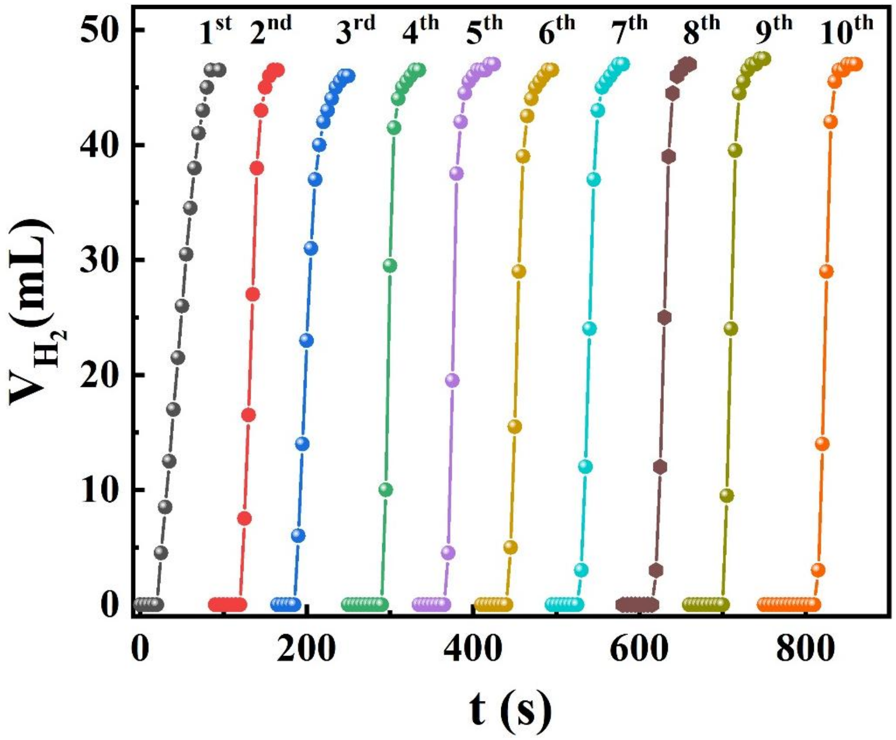

Figure S18. Stability test on the Pt/CNT catalyst in H_2 evolution.

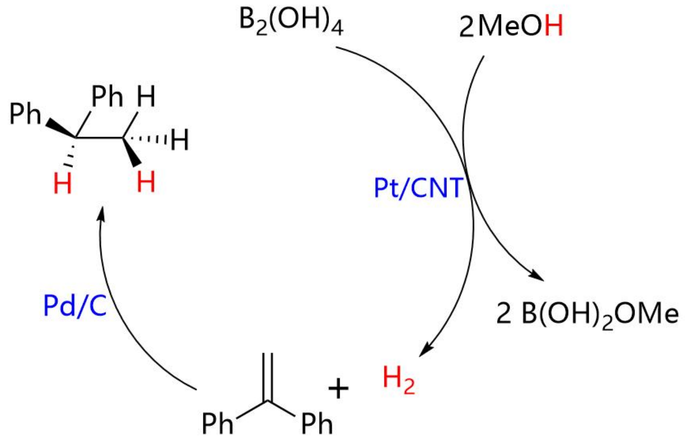

Figure S19. Tandem reaction for 1,1-diphenylethylene hydrogenation.

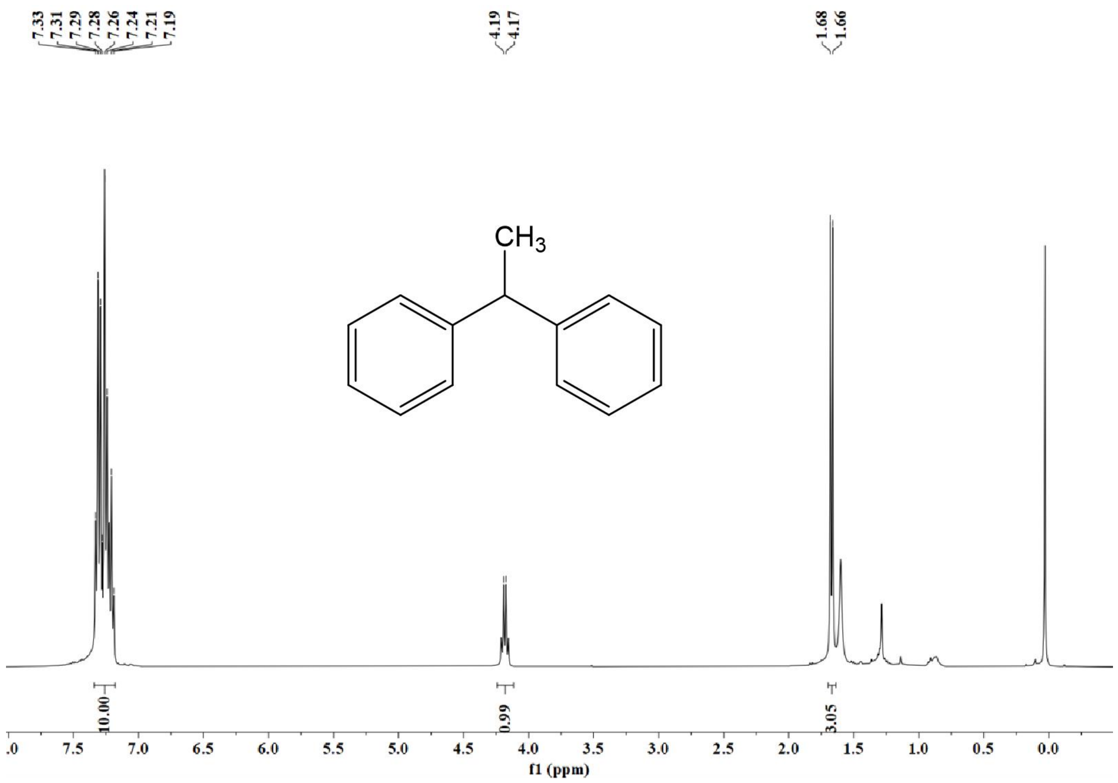

Figure S20. ^{1}H NMR (600 MHz, $CDCl_3$) δ 7.19-7.33 (m,10H), 4.17-4.19 (dd, 1H), 1.66-1.68 (m, 3H).

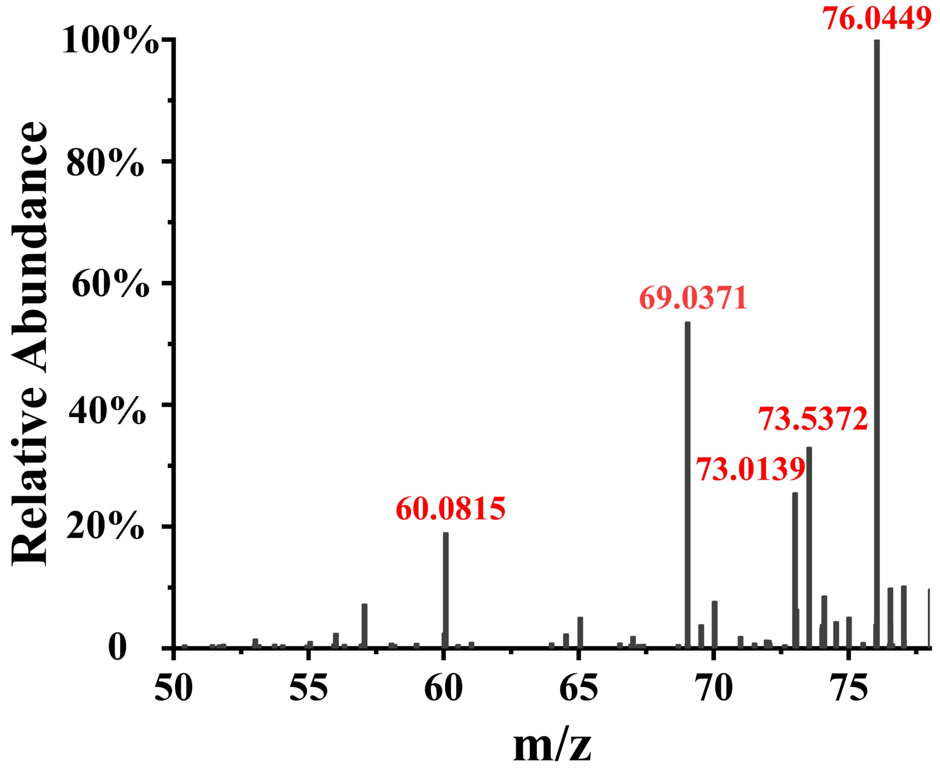

Figure S21. Mass spectrum of $B(OH)_2OMe$. ESI HRMS: calcd. for CH_5BO_3 $[M]^+$: 76.0332, found: 76.0449.

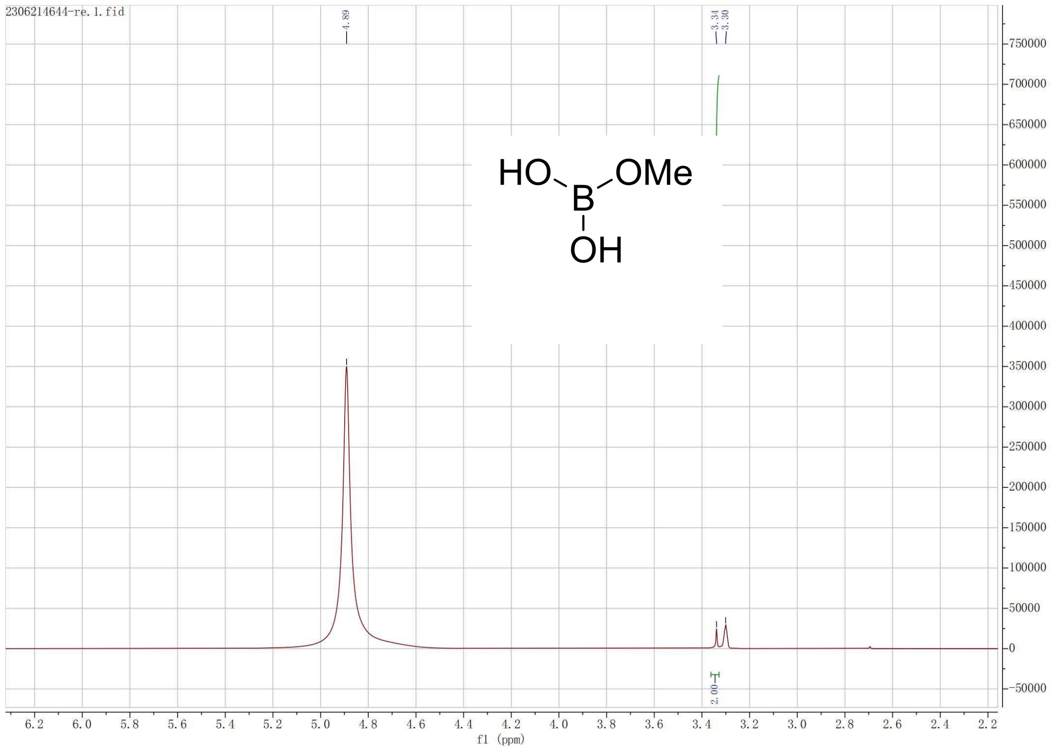

Figure S22. ^{1}H NMR (600 MHz, CD_3OD) δ 4.89 (H_2O), 3.34 (s, 2H), 3.30 (MeOH)

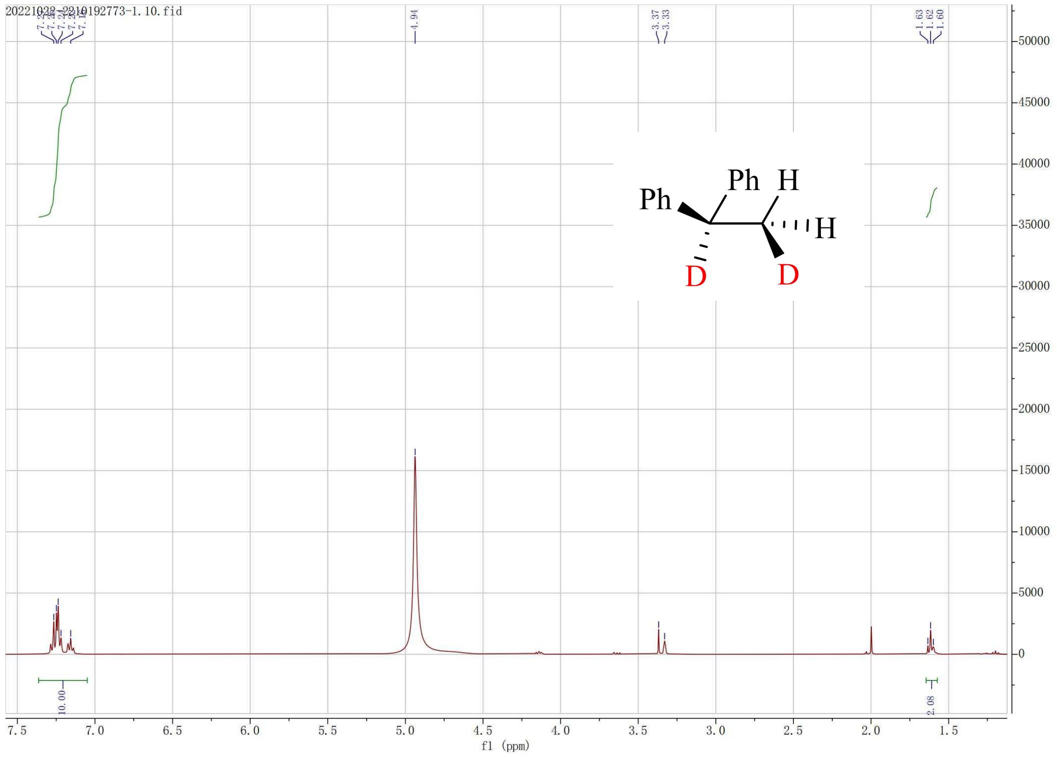

Figure S23. ^{1}H NMR (600 MHz, CD_3OD) δ 7.16-7.27 (m,10H), 1.60-1.63 (m, 2H). Note: δ 4.94 (s, H_2O), δ 3.3 (s, CD_3OD). ref: H. Fujiwara, J. Yamabe, S. Nishimura. *Chem. Phys. Lett*. **2010**, *498*, 42-44

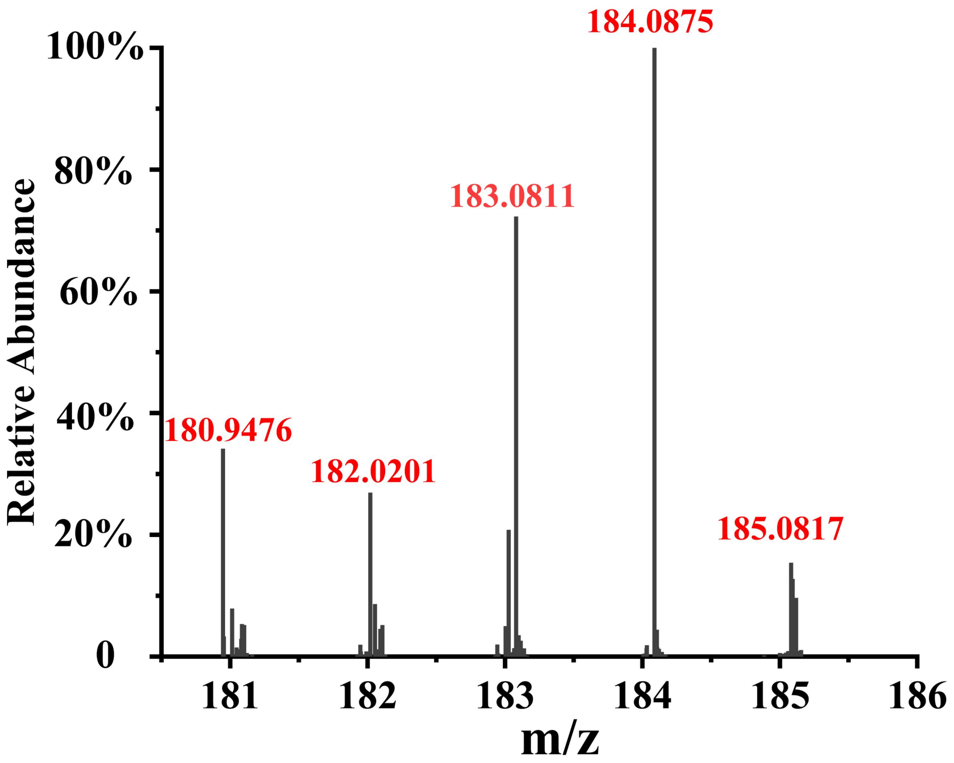

Figure S24. Mass spectrum of deuterated 1,1-diphenylethane. ESI HRMS: calcd. for $C_{14}H_{14}D_2$ $[M+1]^+$: 185.1221, found: 185.0817.

8. Vibrational frequencies in all calculation

$B_2(OH)_4$ vibrations

Mode	Frequency
1	52.92
2	102.83
3	305.63
4	321.54
5	400.23
6	484.00
7	508.06
8	535.27
9	572.53
10	599.95
11	653.64

12	690.93
13	936.78
14	1002.96
15	1003.56
16	1009.96
17	1208.46
18	1332.33
19	1357.99
20	1397.34
21	3793.67
22	3801.41
23	3850.03
24	3851.74

$B_2(OH)_4$ and Pt cluster vibrations

Mode	Frequency
1	24.61
2	47.30
3	71.62
4	80.44
5	93.15
6	97.45
7	98.00
8	103.97
9	116.07
10	141.49
11	171.67
12	183.48
13	199.06
14	209.43

15	321.16
16	352.69
17	367.34
18	423.62
19	490.63
20	541.56
21	574.83
22	611.11
23	632.11
24	682.05
25	986.78
26	1021.84
27	1033.92
28	1064.46
29	1218.97
30	1235.86
31	1266.75
32	1349.07
33	3466.55
34	3610.56
35	3640.51
36	3665.10

$B_2(OH)_4$ adsorbed on Pt cluster vibrations

Mode	Frequency
1	23.12
2	29.52
3	50.01
4	52.66
5	71.48

6	82.81
7	92.97
8	99.03
9	102.19
10	115.30
11	119.66
12	139.30
13	283.56
14	290.78
15	308.44
16	311.94
17	323.77
18	334.58
19	411.82
20	422.57
21	557.48
22	581.63
23	584.43
24	599.52
25	965.11
26	967.48
27	978.83
28	981.77
29	1213.34
30	1220.71
31	1246.15
32	1256.46
33	3731.90
34	3733.51
35	3737.36

36	3745.84

TS vibrations

Mode	Frequency
1	-2948.51
2	13.94
3	24.33
4	31.31
5	53.16
6	67.77
7	75.22
8	82.85
9	89.79
10	91.71
11	96.12
12	110.89
13	120.04
14	123.94
15	133.64
16	199.35
17	249.26
18	273.42
19	306.60
20	435.41
21	440.20
22	488.39
23	498.64
24	587.57
25	601.25
26	632.88

27	673.33
28	701.23
29	710.04
30	746.86
31	767.38
32	778.55
33	928.83
34	939.92
35	976.52
36	987.20
37	1040.64
38	1049.50
39	1070.53
40	1090.08
41	1128.18
42	1158.78
43	1172.22
44	1191.55
45	1217.73
46	1279.49
47	1328.41
48	1384.69
49	1475.59
50	1480.39
51	1482.60
52	1492.63
53	1497.82
54	1501.93
55	1507.72
56	1508.75

57	2572.69
58	2865.29
59	2963.31
60	3090.13
61	3100.67
62	3101.84
63	3203.07
64	3205.97
65	3211.11
66	3211.58
